
JOURNAL INFORMATION
==============================
NLM Title Abbreviation: ScientificWorldJournal
Journal ID: ScientificWorldJournal
NLM Journal ID: 101131163
Journal ID: TSWJ

The Scientific World Journal

ISSN: 2356-6140
EISSN: 1537-744X
Publisher: Wiley

ARTICLE INFORMATION
==============================
PMCID: PMC6009491
PMID: 29973828
DOI: 10.1100/tsw.2002.53
Article ID: 616745
Article version: 1
Subjects: Short Report

Insight into Residues Critical for Antithrombin Function from an Expanded Database of Sequences That Includes Frog, Turtle and Ostrich Antithrombins

Backovic Marija *
Gettins Peter G.W.
Department of Molecular Biology College of Medicine University of Illinois at Chicago, Chicago, IL 60612-4316, USA
*Marija Backovic: mbacko2@uic.edu
Publication date: 2002
Electronic publication date: 2002 Mar 8
Volume: 2
First page: 112
Last page: 114
Copyright: Copyright © 2002 Marija Backovic and Peter G.W. Gettins.
Copyright year: 2002
License: This is an open access article distributed under the Creative Commons Attribution License, which permits unrestricted use, distribution, and reproduction in any medium, provided the original work is properly cited.
License URL: https://creativecommons.org/licenses/by/3.0/

Funding: National Institutes of Health R37 HL49234

==============================
Advances in Gene Technology: The Genome and Beyond –
Structural Biology for Medicine (Proceedings of the 2002 Miami
*Nature Biotechnology* Winter Symposium)
*TheScientificWorld* 2002, 2(S2), 112–114
ISSN 1532-2246; DOI 10.1100/tsw.2002.53

### INSIGHT INTO RESIDUES CRITICAL FOR ANTITHROMBIN FUNCTION FROM AN EXPANDED DATABASE OF SEQUENCES THAT INCLUDES FROG, TURTLE AND OSTRICH ANTITHROMBINS

Marija Backovic* and Peter G.W. Gettins
Department of Biochemistry and Molecular Biology, College of Medicine, University of Illinois at Chicago, Chicago, IL 60612-4316
*mbacko2@uic.edu

**INRODUCTION.** Sequence comparison between homologous proteins from different species is a powerful means of discerning the importance of particular regions or residues in their structure and function, but one that depends on the database of sequences being large enough and diverse enough. Given the importance of antithrombin as an inhibitor of blood coagulation proteinases and as a mediator of antiangiogenic activity we sought to expand the available database by determining the sequences of three antithrombins from diverse families that were either unrepresented or poorly represented in the database of known sequences. We determined antithrombin sequences from frog, turtle, and ostrich, and analyzed conservation of antithrombin, to gain insight into the roles of its particular residues and regions.

**METHOD.** Total RNA was isolated from frozen livers and the initial PCR was performed on oligo(dT)-tailed primed cDNAs. The primers used for the initial PCR were degenerate and designed based on the available antithrombin sequences. Amplified fragments were cloned into pCR2.1-TOPO vector (Invitrogen) and multiple clones were tested for the presence of an insert of the correct size by PCR. The inserts were sequenced in both directions, and gene-specific primers were designed based on the obtained sequences. These primers were used for reading 3' and 5' ends of antithrombin cDNA using 3'RACE and 5'RACE kits (Gibco BRL). All amplification products of correct size were cloned into pCR2.1-TOPO, and DNA from colonies containing inserts of correct size were sequenced in both directions. Complete sequences were assembled using Sequencher 2.0.6 software, and the alignment was carried out using ClustalW[1].

**RESULTS AND DISCUSSION.** Whereas mature human antithrombin is 432 residues long, mature ostrich and turtle antithrombins are each 436 residues long and frog antithrombin is 439 residues long. Overall, human antithrombin shares 72 and 71% identity, respectively, with ostrich and turtle antithrombin. This corresponds to about 120 differences between human and ostrich or turtle antithrombin. Frog antithrombin bears even less identity to human antithrombin, with around 140 amino acids in frog that are substituted by different amino acids in human antithrombin. The addition of three novel antithrombin sequences represent a doubling of the number of sequences that are less than 80% identical to human antithrombin, and decreasing the average identity score from 44 to 39%. This corresponds to 170 amino acids that are intolerant to any changes and share 100% identity among all the species, 123 that are more than 70% identical and 140 that are less than 70% identical.

Sequence comparisons with the other ten known antithrombin sequences and sequences of other serpins (see Fig. 1) has provided striking evidence for the conservation of the heparin activation mechanism and new insight into those residues important for heparin binding, for heparin activation, and for reactive center loop function, as well as an indication of which glycosylation sites might be needed for function. The search for a potential factor Xa exosite needed for enhancement of factor Xa inhibition[2] and for residues important in expressing the anti-angiogenic properties of antithrombin[3] will also be greatly aided by this expanded database.

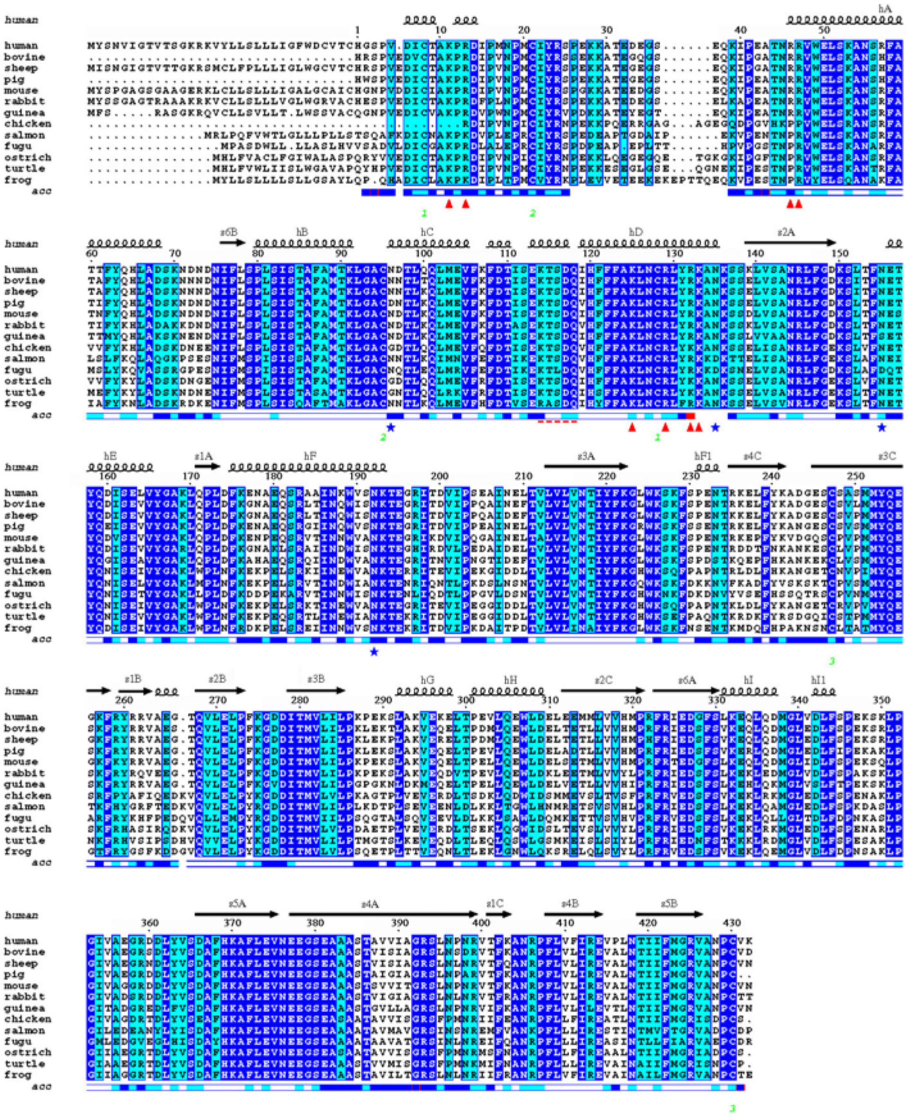

**FIGURE 1**. Aligned sequences of 13 antithrombins including the newly determined sequences of ostrich, turtle, and frog. Sequence numbering is that of mature human antithrombin. Secondary structural elements are based on the structure of native human antithrombin and are shown above the sequences using solid arrows to indicate β-sheet and coils to represent α-helix. Orange triangles indicate basic residues known to be involved in binding heparin. These are highly conserved. Blue stars indicate glycosylation sites in human antithrombin. The three cysteine pairs are identified by green numbers (1, 2, and 3). Positions showing 100% conservation are shown in dark blue, those with conservation between 70 and 100% in light blue and those with less than 70% conservation are uncolored (percentages based on strict conservation). Accessibility, indicated by *acc* below the sequences, is shown as white for buried residues, cyan for partially buried residues and blue for exposed residues (based on the structure of native human antithrombin).

**ACKNOWLEDGMENT.** Supported by grant R37 HL49234 from the National Institutes of Health.

### REFERENCES

1. Thompson, J.D., Higgins, D.G., and Gibson, T.J. (1994) Nucl. Acids Res. 22, 4673–4680.
2. Chuang, Y.-J., Swanson, R., Raja, S.M. and Olson, S.T. (2001) J. Biol. Chem. 276**,** 14961–14971.
3. O'Reilly, M.S., Pirie-Shepherd, S., Lane, W.S. and Folkman, J. (1999) Science 285, 1926–1928.
